# Surgical, functional and audiological evaluation of new Baha^®^ Attract system implantations

**DOI:** 10.1007/s00405-016-3917-5

**Published:** 2016-02-22

**Authors:** Wojciech Gawęcki, Olgierd Maciej Stieler, Andrzej Balcerowiak, Dariusz Komar, Renata Gibasiewicz, Michał Karlik, Joanna Szyfter-Harris, Maciej Wróbel

**Affiliations:** 1 Department of Otolaryngology and Laryngological Oncology, Poznań University of Medical Sciences, Poznań, Poland; 2 KIND Hearing Therapy Centre, Poznań, Poland; 3 Department of Phoniatrics and Audiology, Poznań University of Medical Sciences, Poznań, Poland; 4 Poznań University of Medical Sciences, Poznań, Poland

**Keywords:** Baha^®^ Attract, Bone conduction, Transcutaneous, Bilateral mixed hearing loss, Single-sided deafness, Bone polishing

## Abstract

Bone-anchored hearing aids are well-established solutions for treatment of hearing-impaired patients. However, classical systems with percutaneous abutments have disadvantages concerning aesthetics, hygiene and adverse soft tissue reactions. The study aimed to evaluate surgical, functional and audiological results of a new Baha^®^ Attract system, in which the sound processor is attached by magnetic force. Twenty patients implanted with a Baha^®^ Attract system were divided into two groups: A—bilateral mixed and conductive hearing loss, B—single-sided deafness, and evaluated during a 6-month follow-up. Parameters analysed comprised: (1) surgery and wound healing, (2) postoperative functional results (GBI, APHAB and BAHU questionnaires), (3) audiological results (free field speech in noise audiometry in two situations: with signal from implant side and from contralateral side). Obtained results revealed: mean time of surgery—44 min, soft tissue reduction—30 %, bone polishing—20 %, haematoma—10 %. Functional results showed: GBI total score—29.6 points, APHAB global score mean gain—23.5 %, BAHU ‘good or very good’ score for: aesthetic—85 %, hygiene—100 %, ease of placing the processor—100 %, stability of attraction—75 %. Audiological results—mean gain for the two analysed situations: 32.9 % (group A—36.5 %, group B—27.5 %). To conclude, the data obtained prove the safety and effectiveness of the Baha^®^ Attract system in patients with conductive and mixed hearing loss as well as in patients with single-sided deafness. Cosmetic aspects are highly acceptable and the idea of Attract itself is important for patients with limited manual dexterity.

## Introduction

Bone-anchored hearing aids (BAHAs) are currently well-established solutions for the treatment of hearing-impaired patients with unilateral and bilateral mixed and conductive hearing loss as well as with single-sided deafness. The first implantation was reported by Tjellström and Granström in 1977 [1] and since then more than 100,000 patients have been implanted worldwide [2]. The traditional system is composed of a titanium implant connected with percutaneous abutment and a sound processor which is attached to the abutment. Such a solution enables direct, high-quality sound transmission from the processor to the bone through the abutment and implant. However, percutaneous abutment requires lifelong daily hygienic care and there is also a risk of local skin complications including infections, skin overgrowth and sometimes even implant loss [3]. Additionally, the cosmetic effect is not optimal and some patients who could benefit from the system decline because of the skin-penetrating abutment [4]. Therefore, manufacturers developed a bone conduction device which enables sufficient transmission of the sound with an implanted magnet instead of a percutaneous abutment. The first system, Xomed Audiant, was introduced in the 1980s by Hough et al., but the maximal output was too low for many patients and the system was discontinued after few years [5]. The magnetic Sophono system which was first presented by Siegert under the name Otomag has been available since 2006. It is composed of an implant with two magnets implanted into the temporal bone and a sound processor which is attached outside of the skin by magnetic attraction force [6, 7]. However, due to insufficient amplification it is not indicated in patients with mixed hearing loss with a bone conduction component greater than 45 dB. Recently, new active bone conduction systems with implantable transducers and external sound processors attached by magnetic force have been introduced. The first one—Vibrant Bonebridge (Medel)—is commercially available but it is more expensive and significantly larger than other bone conduction systems [4], and another system BCI is undergoing clinical studies [7].

The system which is going to be studied—Baha^®^ Attract (Cochlear Bone-Anchored Solutions AB, Mölnlycke, Sweden)—was introduced in 2013. It is composed of the same implant and sound processor as a traditional (classical) Baha^®^ Connect system, but instead of percutaneous abutment there are two magnetic discs: one below the skin connected to the implant and a second external one, to which the sound processor is attached. Additionally, a pad of soft material covers the surface of the external magnet and distributes the pressure to the skin and soft tissue between magnets.

The aim of this study is to evaluate the surgical, functional and audiological results of the Baha^®^ Attract system.

## Materials and methods

There are 220 patients who have been implanted since 1992 with the different available systems of bone-anchored solutions in our department. Out of that group, 20 consecutive patients (9.1 %) were implanted with the Baha^®^ Attract (Cochlear Bone-Anchored Solutions AB, Mölnlycke, Sweden) between September 2014 and January 2015. All of those patients were enrolled in this prospective study. The investigation was approved by the local Ethics Committee.

### Group characteristics

The patients (13 female and seven male, aged 25–67 years with a mean of 50) had no history of conditions that could jeopardise osseointegration and wound healing. They were divided into two groups dependent on type of hearing loss: Group A—bilateral mixed or conductive hearing loss—*n* = 12 (*n* = 11 bilateral mixed, *n* = 1 bilateral conductive), and Group B—single-sided deafness—*n* = 8 (*n* = 3 normal hearing in contralateral ear; *n* = 5 mild hearing impairment in contralateral ear). The characteristics of the implanted patients are presented in Table 1. The most frequent indications for the surgery were chronic otitis media (open cavity) and otosclerosis after unsuccessful stapedotomy or restapedotomy.

**Table 1:** Characteristics of the groups of patients

	Group A (*n* = 12)	Group B (*n* = 8)
Audiological indications	Bilateral mixed (*n* = 11) or conductive (*n* = 1) hearing loss	Single-sided deafness (in some cases also mild conductive, mixed or sensorineural hearing loss in the contralateral ear)
Hearing loss	Implanted ear –Mean PTA^a^: 59.1 dB –Mean bone PTA^b^: 30.6 dB	Implanted ear –Mean PTA^a^: 103.6 dB^c^ Contralateral ear –Mean PTA^a^: 31.6 dB –Mean bone PTA^b^: 18.9 dB
Otological indications	Chronic otitis media—7 Atresia of external auditory canal—3 (1 congenital in Treacher Collins syndrome, 2 acquired) Otosclerosis—2	Otosclerosis—6 Post mumps—1 Chronic otitis media—1
Age	53 (36–65)	45 (25–67)
Sex	Male—4 Female—8	Male—3 Female—5

^a^ *PTA* pure-tone average—mean of 500, 1000, 2000 and 3000 Hz

^b^Bone PTA—bone conduction PTA—mean of 500, 1000, 2000 and 3000 Hz in the bone curve

^c^In case of complete deafness (no response) calculated as 120 dB HL

### Surgery and fitting

Surgery was performed in the typical way with a C-shaped incision in all but the first three cases under local anaesthesia. During the 6-month follow-up four ambulatory visits were performed at 10 days, 4 weeks, 3 and 6 months postoperatively. The processor was attached 4 (±1) weeks after surgery. Ten patients received Baha^®^ BP110, eight Baha^®^ 4 and two Baha^®^ 5 processors.

### Evaluated parameters

I Evaluation of surgery and wound healing: The following parameters concerning surgery were analysed: duration of surgery, soft tissue reduction, bone polishing, bipolar coagulation use and any surgical problem or difficulty. The process of healing, cosmetic effect and the patients’ subjective feelings concerning cutaneous sensibility, pain and numbness were also evaluated.
II Functional evaluation: The evaluation was performed 2 months (±1 week) after processor activation. Patients were asked to complete three questionnaires: (1) GBI (Glasgow Benefit Inventory) with additions according to Dutt et al. [8] to evaluate the change in their quality of life after implantation, (2) APHAB (Abbreviated Profile of Hearing Aid Benefit) to evaluate the benefits of the Baha^®^ Attract processor, and (3) BAHU (BAHA Aesthetic, Hygiene and Use) questionnaire to evaluate the patients’ subjective feelings (a newly created, not validated questionnaire, details in Table 5). They were also asked about their mean daily time of use of the Baha^®^ Attract.
III Audiological evaluation: Free field speech in noise audiometry was performed with and without the sound processor 2 months (±1 week) after processor activation. The Polish monosyllabic word test was used. The signal was presented at 65 dB sound pressure level (SPL) from a speaker placed 1 m from the patient on the implant side (Situation 1) and on the contralateral side (Situation 2); white noise was generated from a speaker located 1 m in front of the patient at the 55 dB SPL.

## Results

### Surgery and wound healing

The implantation was performed on the right side in 13 cases and on the left in seven. The mean surgery time (from local anaesthesia to final dressing) was 44 min (range 30–60). There was a need for soft tissue reduction in 30 % of patients (*n* = 6), bone polishing in 20 % (*n* = 4) and bipolar coagulation use in all patients. In one case the bone at the primary implant site was less than 2 mm so second hole was drilled without the need of any additional skin incisions. In one case there was bleeding from an emissary vein, which was closed by a bone wax and the operation was continued.

Healing was uneventful in 90 % (*n* = 18) of cases. In two patients with extensive soft tissue reduction a small haematoma was observed on the day after surgery which was successfully treated by suction and compression during the following days. Mild pain just after surgery was reported by 60 % (*n* = 12) of patients, but after 1 month (second visit) 85 % (*n* = 17) were free from pain. The remaining 15 % (*n* = 3) continued to complain of pain, with significant, gradual decrease in its intensity. 6 months after implantation (last visit) no patient had any pain. The sensitivity of the skin around the implant was normal in 85 % (*n* = 17) of patients; in two it was reduced even 6 months postoperatively and in one there was some numbness which gradually disappeared.

Most of the patients chose magnet number 3 (70 %, *n* = 14) or 4 (25 %, *n* = 5). Only one patient chose magnet number 6. In 85 % (*n* = 17) of patients there was no need to change the initial magnet during follow-up; in two cases it was changed for a weaker magnet (*n* = 1 because of skin redness, *n* = 1 because of pain) and in one it will be changed for a stronger one.

### Functional results

#### Glasgow benefit inventory (GBI)

The results showed significant improvement in health status after implantation (total score 29.6 points) and were similar in both groups (group A—28.0 points, group B—31.9 points). In both groups the highest improvement was observed in the general subscale (total: 40.2 points, group A—38.2 points, group B—43.2 points) and the worst in the physical health subscale (total—3.3, group A—4.2 points, group B—2.1 points). The results of the GBI are presented in Table 2. The results for the first addition introduced by Dutt et al. related to success of BAHA according to patients and their families and friends are presented in Table 3. The second addition, concerning the change in state of health, showed a significant improvement—from 50 % before implantation to 81 % after implantation (*p* < 0.001).

**Table 2:** Quality of life after implantation according GBI (Glasgow Benefit Inventory) questionnaire in both groups of patients (SD—standard deviation)

GBI subscales	Group A		Group B		Group A + Group B	
	Mean	SD	Mean	SD	Mean	SD
Total score	28.0	17.3	31.9	12.9	29.6	15.4
General subscale	38.2	24.2	43.2	17.7	40.2	21.4
Social support	11.1	14.8	16.7	19.9	13.3	16.8
Physical health	4.2	7.5	2.1	5.9	3.3	6.8

**Table 3:** Patients’ subjective opinion regarding success of Baha^®^ Attract

Patient’s opinion	Effectiveness of Baha	Satisfaction with Baha	Effectiveness of Baha in family/friends’ opinion	Recommendation of Baha to others with similar hearing problems
Definitely no	0	0	0	0
Rather no	0	10 %	5 %	0
No change/cannot decide	0	0	5 %	25 %
Rather yes	60 %	55 %	40 %	35 %
Definitely yes	40 %	35 %	50 %	40 %

#### Abbreviated profile of hearing aid benefit (APHAB)

**Fig. 1:**
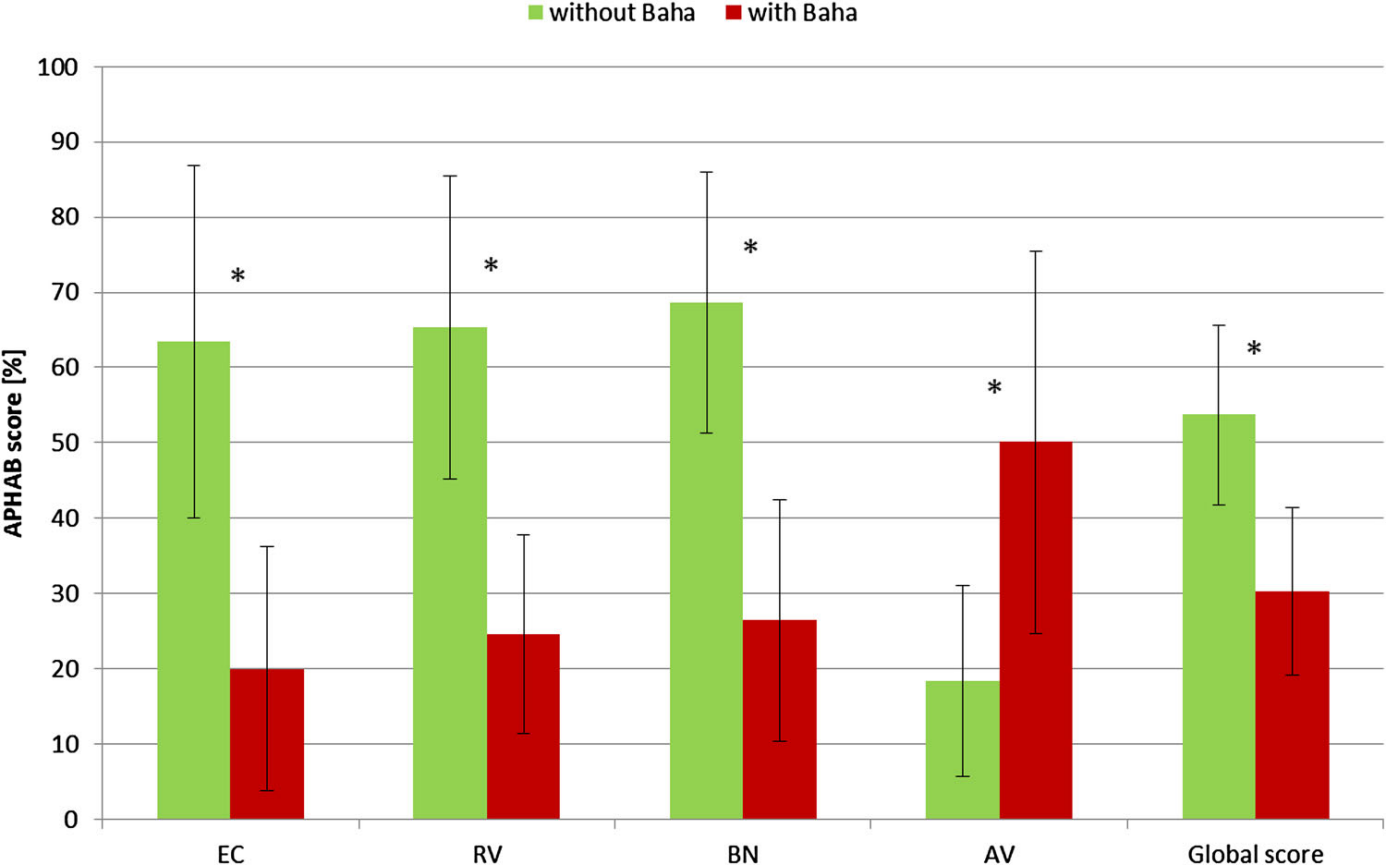
The benefits of the Baha^®^ Attract processor according to APHAB (Abbreviated Profile of Hearing Aid Benefit) questionnaire results; (*n* = 20; *error bars* represent *SD* standard deviation; *p < 0.001, *EC* ease of communication, *RV* reverberation, *BN* background noise, *AV* aversiveness)

In both groups we have observed significant improvement in the Global score (mean gain: total—23.5 %, group A—21.4 %, group B—26.4 %) and in three APHAB subscales: EC (ease of communication) (mean gain: total—43.4 %, group A—40.7 %, group B—47.6 %), RV (reverberation) (mean gain: total—40.8 %, group A—40.2 %, group B—41.5 %), BN (background noise) (mean gain: total—41.5 %, group A—38.1 %, group B—46.5 %), and a significant deterioration in the AV (aversiveness) subscale (mean deterioration: total—31.7 %, group A—32.9 %, group B—29.8 %). The results for the APHAB for all patients are presented in Fig. 1 and results for groups A and B are presented in Table 4.

**Table 4:** Details of APHAB (Abbreviated profile of hearing aid benefit) questionnaire results for both groups of patients (SD—standard deviation)

APHAB subscale	Group A					Group B				
	Without Baha		With Baha		*p*	Without Baha		With Baha		*p*
	Mean (%)	SD (%)	Mean (%)	SD (%)		Mean (%)	SD (%)	Mean (%)	SD (%)	
EC	63.8	22.8	23.1	18.7	<0.005	63.0	25.8	15.4	10.9	<0.02
RV	64.2	20.8	24.0	15.7	<0.005	67.1	20.1	25.6	9.3	<0.001
BN	65.2	16.9	27.1	16.7	<0.001	72.0	18.0	25.5	16.2	<0.02
AV	21.1	10.4	54.0	26.4	<0.005	14.3	15.4	44.1	24.2	<0.05
Global score	53.5	11.4	32.1	11.8	<0.005	54.1	13.4	27.7	10.1	<0.001

*EC* ease of communication, *RV* reverberation, *BN* background noise, *AV* aversiveness

#### BAHA aesthetic, hygiene and use (BAHU)

All patients found it easy or very easy to place the Baha^®^ Attract system processor on the head and it caused no or only mild hygiene problems in the operated area. Eighty-five percent (*n* = 17) of patients answered that the aesthetic effect of the system is very good or good. Only one man—a completely bald teacher in secondary school—was not satisfied with the aesthetic effect of the system as the processor is visible to his students. There were no problems with the stability of processor attraction on the head in 75 % (*n* = 15) of cases. However, one woman with magnet number 3 complained to have such a problem every day so the magnet will be changed for a number 4 soon. The results of BAHU for all patients are presented in Table 5.

**Table 5:** Patients’ subjective feelings concerning Baha^®^ Attract according BAHU (BAHA aesthetic, hygiene and use) questionnaire (new, not validated)

Scale of patient’s feelings	Aesthetic aspect	Hygiene	Ease of placing the processor	Stability of the attraction
1—very negative	0	0	0	0
2—negative	5 %	0	0	5 %
3—neutral	10 %	0	0	20 %
4—positive	25 %	10 %	10 %	35 %
5—very positive	60 %	90 %	90 %	40 %

The mean daily time of use of the Baha^®^ Attract was 9.6 h (range 2–16 h) and it was similar in group A (mean 10 h, range 2–16 h) and group B (mean 9 h, range 5–15 h).

#### Audiological results

**Fig. 2:**
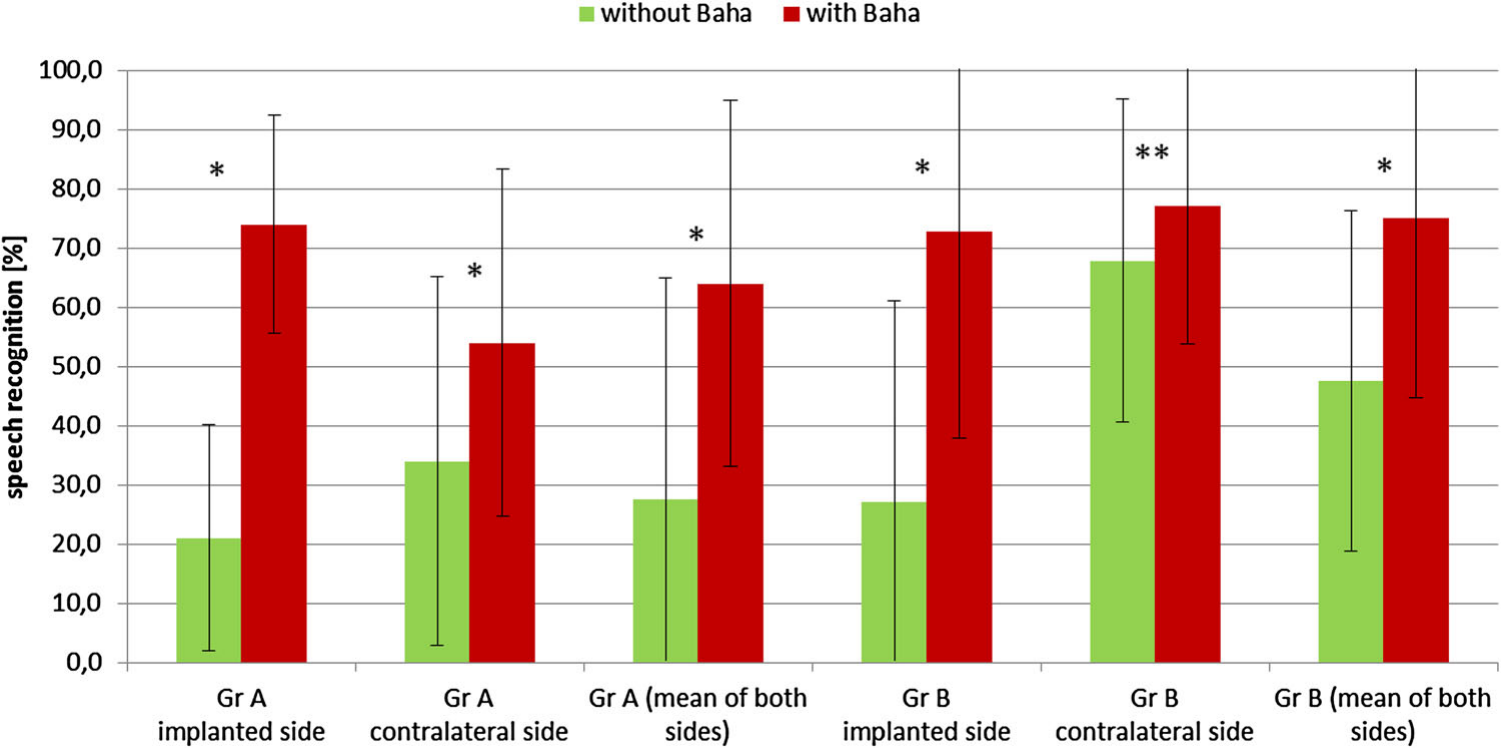
Audiological results—free field speech in noise audiometry with and without Baha^®^ Attract (*n* = 17, *error bars* represent *SD* standard deviation; *p < 0.001, **p < 0.05)

The audiological examination was performed in 17 patients (ten from group A and seven from group B). In Situation 1 in both groups a significant improvement of speech understanding in noise was observed (mean gain: total—50.0 %, group A—53.0 %, group B—45.8 %). In Situation 2 improvement was not so evident (mean gain: total—15.7 %, group A—20.0 %, group B—9.3 %). The mean value for both situations was also calculated: the mean gain in all patients was 32.9 %, in group A—36.5 % and in group B—27.5 %. The audiological results are presented in Fig. 2.

## Discussion

Devices which use bone conduction have been implanted with success for many years and percutaneous BAHA implants are accepted as the gold standard [2]. Their efficacy is well proved but unfortunately they have some important disadvantages effected by percutaneous abutment. Implantation of a BAHA is generally a safe procedure with a very limited number of serious complications; however, the number of soft tissue problems around the abutment (soft tissue overgrowth and abutment side infection) is frequent [9]. Some changes in the operation technique have been introduced to limit the disadvantages of percutaneous abutments (soft tissue preservation, pouch technique) but systems without percutaneous abutment seem to be the best option for cosmetic and hygienic concerns and the state of the soft tissue. However, such magnetic systems can cause different problems like limited transmission of vibrations through the skin, especially at high frequencies, and problems with good retention. Proper construction of systems which allow limitation of pressure on soft tissue, and development of modern processor technology which makes it possible to compensate for skin attenuation by increasing the amplification in affected frequencies seems to be crucial to prevent these problems [2, 10].

Surgical results for Baha^®^ Attract were previously reported by Iseri et al. [2, 11] and by Briggs et al. [12]. The mean surgery time in those studies was very similar to that reported in this study: 48 min [2], 46 min [11] and 45 min [12]. The operations were performed under general anaesthesia [2, 12] or under local or general anaesthesia according to the patient’s preference [11]. In our study 85 % of patients were operated on under local anaesthesia without any problems and we think that Baha^®^ Attract surgery in most adults can be performed this way. Soft tissue reduction was performed in five of 16 patients (31.2 %) [11] and three of 27 were operated on (11.1 %) [12]. In our group it was performed in six of 20 patients (30 %), placing our patients within the same range. The need for bone polishing was reported only in one paper and was performed in five of 12 (41.6 %) [2] and it was higher than in our study (four of 20, 20 %). Good healing was observed in all reported cases to date. Iseri et al. described one case of haematoma on the first postoperative day which was successfully treated by aspiration, one case of temporary skin erythema with pain and three cases of pain around the implant which gradually disappeared after decreasing implant strength [2, 11]. Briggs et al. described four cases of mild erythema (three dissolved without treatment, one dissolved after changing the magnet for a weaker one), four cases of pain on the implant side (two resolved without treatment, two were mild and present for a longer time) and one patient with some discomfort on the implant side which resolved without treatment [12]. They observed a lot of numbness at the time of initial fitting (4 weeks—62.9 %) which then gradually decreased (3 months—25.9 %), and in the majority of patients there was no pain or pain was very limited [12]. In their group most of the patients chose magnet number 5 (17 of 27 patients, 63 %) and more than half of patients required a change of magnet strength, most of them for a weaker one [12]. In our group, healing was uneventful in 90 % of cases and there was no need to change the initial magnet in 85 %, but our follow-up is relatively short so further observations of operated area and the need to change the magnet are necessary.

The GBI results after Baha^®^ Attract implantation were presented and compared to patients with percutaneous Baha^®^ Dermalock by Iseri et al. [11]. In the Baha^®^ Attract group they observed an improvement in total score (40.5 points) and in all subscales: General (47.6 points), Social Support (28.1 points) and Physical Health (23.9 points). These outcomes were similar to those for patients implanted with a Baha^®^ Dermalock [11]. The results for our group also showed improvement in the total score (29.6 points) and all subscales: General (40.2 points), Social Support (13.3 points) and Physical Health (3.3 points) but this improvement is slightly smaller than those presented by Iseri et al. It can be explained by the different population examined (Polish vs. Turkish) and by the type and depth of hearing loss—Iseri et al. described only patients with bilateral conductive or primarily conductive hearing loss and our group contains patients mainly with mixed hearing loss (mean bone pure-tone average 30.6 dB) and with single-sided deafness. The outcomes for our group are very similar to results of the Polish population implanted with percutaneous BAHAs (multicentre national study, unpublished data) for total score (31.9 points) and all subscales: General (43.6 points), Social Support (15.4 points) and Physical Health (1.71 points).

The outcomes for APHAB in patients with Baha^®^ Attract were presented by Briggs et al. [12]. They found statistically significant improvement for the APHAB Global score and Reverberation and Background Noise subscales, nonsignificant improvement for the Ease of Communication subscale, and nonsignificant deterioration for the Aversiveness subscale. The improvement in the Global score is 16 %, Background Noise 17 %, Ease of Communication 12 % and the deterioration in Aversiveness 12 %. In our group we have observed an even higher benefit after implantation in the Global score (23.5 %) and Ease of Communication (43.4 %), Reverberation (40.8 %) and Background Noise (41.5 %) subscales, and deterioration in the Aversiveness subscale (31.7 %). Such a deterioration in this last subscale which quantifies negative reactions to environmental sounds is typically observed with different hearing devices because unwanted sounds also are amplified [13].

The results for the BAHU scale suggest that the Baha^®^ Attract, in the opinion of most of patients, is very aesthetic, easy to maintain hygienically, it is easy to place the processor on the head and has good stability of attraction.

The mean daily time of use of Baha^®^ Attract reported by Briggs et al. [12] was 7.0 h (range 3.4–15.4 h). It was longer in patients with conductive hearing loss (mean 7.6 h) than in single-sided deafness (mean 6 h). In our group the average time of daily use was longer—9.6 h (range 2–16 h), and it was a little longer in group A (mean 10 h) than in group B (mean 9 h). Such a high mean daily use of the device may suggest good efficacy and good wearing comfort in most patients.

Audiological results for Baha^®^ Attract were previously presented by Iseri et al. (patients with bilateral conductive or primarily conductive hearing loss) [2, 11] and by Briggs et al. (patients with conductive or mild mixed hearing loss and with single-sided deafness) [12]. Those studies, however, did not include patients with mixed hearing loss with mean a bone conduction threshold worse than 30 dB. Iseri et al. showed improvement of the free field hearing threshold from 45 dB without Baha^®^ Attract to 37 dB with Baha^®^ Attract, and the free field speech recognition threshold from 56 dB without Baha^®^ Attract to 37 dB with Baha^®^ Attract [2]. In the next paper they compared audiological outcomes between Baha^®^ Attract and a percutaneous system. This study showed the benefit of both systems, but the results for the frequency-specific hearing threshold in free field and speech reception thresholds showed a better gain for the percutaneous system especially for speech reception thresholds and in high-frequency hearing thresholds [11]. Similarly, Briggs et al. [12] presented a statistically significant improvement of pure-tone average, speech recognition in quiet and speech-to-noise ratio (SNR) in adaptive sentence test in noise after Baha^®^ Attract implantation. In all these tests the results were similar to the softband test. In our study significant improvement of speech understanding in noise was observed in both groups analysed—the mean gain of the two analysed situations in group A was 36.5 % and in group B 27.5 %. The results for group A are similar to results for the Polish population implanted with percutaneous BAHAs and analysed under the same conditions (multicentre national study, unpublished data) in patients with bilateral mixed hearing loss (38.3 %) and bilateral conductive hearing loss (34.7 %), and the results for group B are even better than in patients with single-sided deafness implanted with a percutaneous BAHA (16.1 %).

## Conclusions

Implantation of the Baha^®^ Attract system is an easy, safe and effective procedure. It can be performed under local anaesthesia in adults. There are no major surgical problems or complications and in most patients healing, final cosmetic effect and wearing comfort are very good. The functional and audiological results show significant gain after implantation in patients with conductive and mixed hearing loss as well as those with single-sided deafness. The Baha^®^ Attract is a good alternative to percutaneous systems especially for patients for whom the aesthetic aspect is important and for patients with limited manual dexterity.
